# Development of a Multiplex Real-Time PCR for the Rapid Detection of the Predominant Beta-Lactamase Genes CTX-M, SHV, TEM and CIT-Type AmpCs in Enterobacteriaceae

**DOI:** 10.1371/journal.pone.0100956

**Published:** 2014-07-17

**Authors:** Nicole Roschanski, Jennie Fischer, Beatriz Guerra, Uwe Roesler

**Affiliations:** 1 Institute of Animal Hygiene and Environmental Health, Free University Berlin, Berlin, Germany; 2 Federal Institute for Risk Assessment, Department of Biological Safety, Berlin, Germany; Charité-University Medicine Berlin, Germany

## Abstract

Beta-lactamase resistant bacteria and especially ESBL producing Enterobacteriaceae are an increasing problem worldwide. For this reason a major interest in efficient and reliable methods for rapid screening of high sample numbers is recognizable. Therefore, a multiplex real-time PCR was developed to detect the predominant class A beta-lactamase genes *bla*_CTX-M_, *bla*_SHV_, *bla*_TEM_ and CIT-type AmpCs in a one-step reaction. A set of 114 Enterobacteriaceae containing previously identified resistance gene subtypes and in addition 20 undefined animal and environmental isolates were used for the validation of this assay. To confirm the accessibility in variable settings, the real-time runs were performed analogous in two different laboratories using different real-time cyclers. The obtained results showed complete accordance between the real-time data and the predetermined genotypes. Even if sequence analyses are further necessary for a comprehensive characterization, this method was proofed to be reliable for rapid screening of high sample numbers and therefore could be an important tool for e. g. epidemiological purposes or support infection control measures.

## Introduction

In Gram-negative bacteria, the production of beta-lactamases represents the most important contributing factor to resistance against beta-lactam antibiotics. During the last couple of years increasing numbers of antibiotic-resistant bacteria have become a problem in the field of infection control. Particularly in Enterobacteriaceae, extended-spectrum- and AmpC-type beta-lactamases play an important role [1]–[3]. Extended-spectrum beta-lactamases (ESBLs) have the ability to hydrolyze various types of the newer beta-lactam antibiotics, including extended-spectrum cephalosporins of the 3^rd^ and 4^th^ generation (e.g. cefotaxime, ceftriaxone, ceftazidime) and monobactams (e.g. aztreonam), which were assessed as “critically important antimicrobials” by the WHO [4]. Nowadays the predominant ESBL-gene families encountered are *bla*_CTX-M_, *bla*_TEM_ and *bla*_SHV_ [5]. Until now 219 different TEM- and 186 SHV- sequences have been published within the Lahey database (http://www.lahey.org/Studies/; June 20^th^, 2014). While the DNA sequences of *bla*_TEM_ and *bla*_SHV_ are very homologues and just a few point mutations at selected loci within the gene give rise to the ESBL-phenotype [6], the *bla*_CTX-M_ genes show a bigger range of variability. So far 157 CTX-M variants have been described (http://www.lahey.org/Studies/; June 20^th^, 2014) which can be divided into five different groups, based on their amino acid sequence identities. These subgroups are specified as CTX-M-1/group; CTX-M-2/group; CTX-M-8/group; CTX-M-9/group and CTX-M-25/group among which the groups CTX-M-1, -M-2 and –M-9 are the predominant ones [7].

Also, the plasmid-encoded AmpC cephalosporinases can be arranged into four general categories among which the CIT-type AmpC beta-lactamases (e.g. CMY-2,-3,-4, LAT-1, LAT-2, BIL-1) are commonly detected [8]–[10]. Especially the CMY-2 type is the most frequently recovered AmpC beta-lactamase from patients in hospitals and in the community, as well as from livestock and ground meat [11] and therefore it is described as the most common plasmid-mediated AmpC beta-lactamase worldwide [12].

As the horizontal plasmid transfer is an important factor for the epidemiology of the resistance genes within the bacterial ecosystem, rapid and cost-effective methods for identification of these genes are necessary. Particularly in the field of food and livestock associated bacteria (zoonotic as well as commensal ones) the monitoring of antimicrobial resistance (ESBL, AmpC and Carbapenemases) becomes more and more important and quite recently the European (EU) legislation has been revised and specific monitoring programs are obliged for all EU member states [13].

Until now, a common method for the phenotypical confirmation of ESBL-/AmpC producing bacteria is the use of susceptibility testing and the following interpretation of the obtained results by harmonized criteria e.g. the CLSI- [14] and EUCAST guidelines [15]. But it has to be considered that the development of resistance is dependent on the mode and level of expression and furthermore it cannot be excluded that resistance genes which are not expressed *in-vitro* may show an expression in *in-vivo* surroundings [16]. Moreover, resistant strains often contain more than one ESBL and/or AmpC gene belonging to different resistance gene families. This can lead to an interference with the result of the susceptibility testing. Therefore, a reliable identification and characterization of the beta-lactamase producers often requires genotypic verification [1]. For this reason a multiplex real-time PCR was developed to detect the predominant class A beta-lactamase genes *bla*_CTX-M_, *bla*_SHV_, *bla*_TEM_ and CIT-type AmpCs in a one-step reaction. To validate the assay and make it widely accessible to diagnostic laboratories a set of 114 bacterial isolates containing previously identified resistance gene subtypes were tested. To survey the reproducibility of the obtained results, all of the real-time runs were performed analogous in two different laboratories using either the Roche Lightcycler 480II or the BioRad CFX96.

## Materials and Methods

### 1. Bacterial strains

Bacterial strains used in this study originated from different sources (livestock and companion animals, animal housings and environment as well as a few isolates from food and humans or reference strains).

A set of 114 control isolates derived either from the strain collection of the National Reference Laboratory for Antimicrobial Resistance (Federal Institute for Risk Assessment, Berlin, Germany) or were taken from the strain collection of the Institute of Animal Hygiene and Environmental Health (Free University (FU-) Berlin, Germany) which includes the majority of the livestock associated ESBL-isolates of the German research consortium RESET (www.reset-verbund.de). The CTX-M-26 positive isolate was kindly provided by the Institute of Microbiology and Epizootics (FU-Berlin, Germany) and six strains carrying different genes (*bla*_TEM_, *bla*_CTX-M_; see Table 1) were made available by the European Reference Laboratory for Antimicrobial Resistance (EURL-AR, Lyngby, Denmark). In addition two *Klebsiella pneumoniae* isolates containing the *bla*_SHV-11_ were provided by the Robert-Koch-Institute (Wernigerorde, Germany).

**Table 1:** Overview of the class A and class C beta-lactamase gene subtypes present within the 114 bacterial isolates used for the assay validation.

beta-lactamase family	Sequence subtype	Organism
***bla*** **_TEM_**	TEM-1	*E. coli* (n = 25), *Salmonella* spp. (n = 12) *Klebsiella* spp. (n = 3)
	TEM-20	*Salmonella* spp. (n = 3)
	TEM-30^a^	*E. coli* (n = 2)
	TEM-37^a^	*E. coli* (n = 1)
	TEM-52	*E. coli* (n = 6), *Salmonella* spp. (n = 6)
	TEM-63^a^	*Salmonella* spp. (n = 1)
	TEM-116	*Salmonella* spp. (n = 1)
	TEM-127^a^	*E. coli* (n = 1)
	TEM-128^a^	*E. coli* (n = 1)
	TEM-135	*E. coli* (n = 1)
	TEM-206	*E. coli* (n = 1)
***bla*** **_SHV_**	SHV-2	*Salmonella* spp. (n = 2)
	SHV-11^b^	*Klebsiella* spp. (n = 3)
	SHV-12	*Salmonella* spp. (n = 3), *E. coli* (n = 4)
***bla*** **_CTX-M-1/group_**	CTX-M-1	*E. coli* (n = 5), *Salmonella* spp. (n = 5)
	CTX-M-3	*Salmonella* spp. (n = 1)
	CTX-M-15	*E. coli* (n = 4), *Salmonella* spp. (n = 2), *Klebsiella* spp. (n = 1)
	CTX-M-22	*Salmonella spp.* (n = 1)
	CTX-M-28^a^	*Salmonella* spp. (n = 1)
	CTX-M-55	*Salmonella* spp. (n = 1)
***bla*** **_CTX-M-2/group_**	CTX-M-2	*E. coli* (n = 5), *Salmonella* spp. (n = 3)
***bla*** **_CTX-M-8/group_**	CTX-M-8	*Salmonella* spp. (n = 1)
***bla*** **_CTX-M-9/group_**	CTX-M-9	*E. coli* (n = 1), *Salmonella* spp. (n = 1)
	CTX-M-14	*E. coli* (n = 2), *Salmonella* spp. (n = 1)
	CTX-M-24	*E. coli* (n = 3)
***bla*** **_CTX-M-25/group_**	CTX-M-26^c^	*Klebsiella* spp. (n = 1)
***bla*** **_AmpC-CIT_**	CMY-2	*E. coli* (n = 6), *Salmonella* spp. (n = 6)
	CMY-16	*Salmonella* spp. (n = 1)
**negative controls**	none of the four beta-lactamase genes	*E. coli* (n = 22), *Salmonella* spp.(n = 1)

Within this table the previously notified sequence information was corrected by the new screening results shown in table 5.

n = number of organisms.

a Isolates provided by the EURL-AR Reference Laboratory (Lyngby, Denmark).

b Two of the three isolates were provided by the Robert Koch Institute (Wernigerode, Germany).

c Isolate provided by the FU-Berlin - Institute of Microbiology and Epizootics (Berlin, Germany).

All of the control isolates were previously characterized by their particular supplier laboratories (mentioned above) and the sequence subtype of each detected resistance gene was known. Ninety-one of these isolates were positive for at least one of the four resistance gene families tested in which all of the five CTX-M subgroups were covered. A total of 56 strains contained one of the four beta-lactamase genes, 32 isolates possessed two resistance genes in different combinations and the remaining three strains showed positive results for all of the tested class A beta-lactamase genes (*bla*_TEM_, *bla*_SHV_ and *bla*_CTX-M_). Twenty-three strains showed none of the tested resistance genes in conventional PCR formats and therefore served as negative controls for the establishment of the real-time PCR assay. Detailed information about the different types of beta-lactamase resistance genes which were covered within this study is shown in Table 1.

Moreover 20 animal and environmental isolates were used as undefined (“blind”) test strains for the final validation of the assay. At this juncture a set of different bacteria was used: seven *Klebsiella pneumoniae* isolates, eight *Escherichia coli* strains, three *Salmonella* spp. and one isolate of *Proteus mirabilis* and *Acinetobacter* spp. each.

### 2. DNA extraction

Bacterial isolates were grown in liquid medium overnight. 1 mL of each culture was taken and pelletized at 14,000 rpm for 3 min. The remaining cell pellet was resuspended in 300 µL sterile TE-buffer (10 mM Tris, 0,1 mM EDTA, pH 8) and heated to 98°C for 10 min. Afterwards the suspension was cooled down on ice and the cell debris was removed by a 2 min. centrifugation step (14.000 rpm). The remaining supernatant, containing chromosomal as well as plasmid DNA fractions, was directly used for PCR reactions. For further experiments the samples were stored at −20°C.

### 3. Primer and TaqMan Probes

Detailed information about the already characterized beta-lactamase genes were obtained from the Lahey database (http://www.lahey.org/Studies/). To ensure that the primer/probe combinations for each beta-lactamase gene family detect the corresponding gene subtypes, a set of up to 25 different sequences was applied to generate an alignment feasible as basis for the primer/probe design.

Sequence data of the genes were downloaded from the GenBank web site (http://www.ncbi.nlm.nih.gov/genbank/) and aligned using the Lasergene DNA software package 10 (DNAStar, Madison, USA).

One exception was represented by the diverse group of CTX-M genes. In this case it was not possible to cover all of the five subgroups using just one set of primers/probe. For this reason the five groups were combined to two major clusters containing either the groups CTX-M-1 and –M-9 (Cluster A) or the groups CTX-M-2, -M-8 and -M-25 (Cluster B). For each of the two clusters a separate set of primers and probes was developed. Never the less, both of the CTX-M probes were labeled with the same fluorophor (Yakima Yellow) to ensure the detection of all five CTX-M groups in one channel of the real-time cycler.

The used primers and probes were synthesized by biomers.net GmbH (Ulm, Germany). Sequences and references of the primer pairs are summarized in Table 2.

**Table 2:** Primers and probes used in this study.

Target	Primer−/Probe- name	Sequence (5′- 3′)	Reference
**Real-time PCR:**			
***bla*** **_TEM_**	TEM_fwd.	GCATCTTACGGATGGCATGA	this work
	TEM_rev.	GTCCTCCGATCGTTGTCAGAA	
	TEM_probe	**6-Fam**-CAGTGCTGCCATAACCATGAGTGA-**BHQ-1**	
***bla*** **_CMY_**	CMY_fwd.	GGCAAACAGTGGCAGGGTAT	this work
	CMY_rev.	AATGCGGCTTTATCCCTAACG	
	CMY_probe	**ROX**-CCTACCGCTGCAGATCCCCGATG-**BHQ-2**	
***bla*** **_SHV_**	SHV_fwd.	TCCCATGATGAGCACCTTTAAA	this work
	SHV_rev.	TCCTGCTGGCGATAGTGGAT	
	SHV_probe	**Cy5**-TGCCGGTGACGAACAGCTGGAG-**BBQ-650**	
***bla*** **_CTX-M_**	CTX-A_fwd.	CGGGC**R**ATGGCGCA**R**AC	this work
	CTX-A_rev.	TGC**R**CCGGT**S**GTATTGCC	
	CTX-A_probe	**Yakima Yellow**-CCA**R**CGGGCGCAG**Y**TGGTGAC-**BHQ1**	
	CTX-B_fwd.	ACCGAGCC**S**ACGCTCAA	
	CTX-B_rev.	CCGCTGCCGGTTTTATC	
	CTX-B_probe	**Yakima Yellow**- CCCGCG**Y**GATACCACCACGC-**BHQ1**	
**Conventional PCR and sequencing:**			
***bla*** **_TEM_**	TEM-F	GCGGAACCCCTATTTG	[24]
	TEM-R	ACCAATGCTTAATCAGTGAG	
***bla*** **_SHV_**	SHV-F	TTATCTCCCTGTTAGCCACC	[25]
	SHV-R	GATTTGCTGATTTCGCTCGG	
***bla*** **_CMY_**	CMY-F	ATGATGAAAAAATCGTTATGCT	[26]
	CMY-R	TTATTGCAGCTTTTCAAGAATGCG	
***bla*** **_CTX-M_**	CTX-M universal_F	CGATGTGCAGTACCAGTAA	[27]
	CTX-M universal_R	TTAGTGACCAGAATCAGCGG	
	CTX-M-1_Seq_F	CCCATGGTTAAAAAATCACTGC	[28]
	CTX-M-1_Seq_R	CAGCGCTTTTGCCGTCTAAG	

### 4. Multiplex real-time PCR

Real-time amplifications were performed in 25 µL reactions containing 12.5 µL ABsolute qPCR Mix (Thermo Scientific, St. Leon Roth, Germany), 1 µL of each forward and reverse primer (10 pmol), 0.1 µL TEM TaqMan probe (5 pmol), 0.2 µL of each of the other four TaqMan probes (10 pmol), 0.6 µL of sterile water and 1 µL of DNA-mixture. If the boiled cells were stored at −20°C for several months, it could have been necessary to extend the DNA amount up to 2 µL to generate improved real-time results.

To figure out the optimal real-time PCR conditions and to confirm the specificity of the assay, all of the five primer/probe pairs were investigated by using strains containing only one single resistance gene for the particular primer/probe combination. For this purpose a set of five positive control strains (P1–5) plus one negative control strain (N1), which was known to contain none of the four tested resistance genes, were chosen from the set of the 114 previously characterized control strains (Table 3). In addition the obligatory “no template control” (NTC) was part of every single real-time run. To proof the functionality of the assay in variable settings the real-time runs were performed analogous in two different laboratories using either the Lightcycler480II (Roche Diagnostics GmbH, Mannheim, Germany) or the CFX96 (Bio Rad Laboratories GmbH, Munich, Germany).

**Table 3:** Validation of the real-time PCR assay.

			Roche Lightcycler 480II				BioRad CFX96			
			Average over six technical replicates				Average over six technical replicates			
			Standard deviation				Standard deviation			
Strain	*bla*- gene	Primer/probe combination	465–510nm	618–660nm	533–610nm	533–580nm	Channel1: FAM	Channel4: Cy 5	Channel3: ROX	Channel2: VIC
NTC			none				none			
N_1_	-		none				none			
		CMY_fwd.			13.0				14.7	
P_1_	CMY-2	CMY_rev.			1.7				2.2	
		CMY_probe								
		CTX-A_fwd				14.7				15.1
P_2_	CTX-M-15	CTX-A_rev.				2.3				3.4
		CTX-A_probe								
		CTX-B_fwd.				15.3				16.3
P_3_	CTX-M-8	CTX-B_rev.				2.0				1.3
		CTX-B_probe								
		SHV_fwd.		14.7				15.7		
P_4_	SHV-12	SHV_rev.		2.2				2.4		
		SHV_probe								
		TEM_fwd.	15.4				14.5			
P_5_	TEM-52	TEM_rev.	2.8				4.6			
		TEM_probe								

For the assay validation one of the 23 negative control strains (N1) and five of the 91 positive control strains (P1–P5), known to possess one of the beta-lactamase resistance genes that should be accounted within this assay, were used. The runs were performed in six technical replicates and the mean C_t_ values for each primer/probe combination used in this multiplex assay are indicated.

NTC = No Template Control.

The real-time conditions were chosen as follows: To achieve a maximum of polymerase activity a preliminary heating step at 95°C for 15 min was necessary. This was followed by 30 cycles of: 95°C for 15 sec; 50°C for 15 sec and 70°C for 20 sec. Fluorescence signals were detected in four different channels: Green (465–510 nm)/6FAM, Orange (533–610 nm)/ROX, Red (618–660 nm)/Cy5 and Yellow (533–580 nm)/Yakima Yellow. To prevent a color-crosstalk between the four different channels of the Roche Lightcycler, it was furthermore necessary to perform a color-compensation run according to the instruction described in the Lightcycler 480II manual. These data were saved and automatically compared to each of the performed runs to get unambiguous results.

After completion of the run, a cycle threshold (C_t_) was calculated by determining the signal strength at which the fluorescence exceeded a threshold limit. This value was manually set for each detection channel and each experiment. Samples possessing a fluorescence signal above this value were assessed as positive.

### 5. Conventional PCR and sequence analyses

To proof the received real-time results of the “blind samples”, conventional PCR formats followed by the sequencing of the PCR products were carried out. Therefore the primers shown in Table 2 (“Conventional PCR and sequencing”) were used. PCR amplifications were performed in 50 µL reactions containing 25 µL DreamTaq Green PCR Master Mix (Thermo Scientific, St. Leon Roth, Germany), 2 µL of each forward and reverse primer (10 pmol), 20 µL of sterile water and 1 µL of DNA-mixture.

The PCRs were performed with an initial denaturation step of 95°C followed by 35 cycles – denaturation at 95°C for 30 sec, annealing for 30 sec and elongation for 1 min. at 72°C – before finishing the run a final elongation step at 72°C for 5 min was attached. Amplified PCR fragments were purified for sequencing using the innuPREP PCRpure Kit (Analytik Jena, Jena, Germany). Sequencing of the PCR products was conducted by LGC Genomics (Berlin, Germany). The obtained sequences were analyzed using the program ‘‘SeqMan Pro’’ of the Lasergene10 Core Suite (DNASTAR, Inc., Madison, USA).

## Results

### Validation of the real-time assay

The described multiplex real-time PCR assay was validated using five of the 91 isolates, known to possess one of the accounted beta-lactamase resistance genes (P1–5; see Table 3). Each isolate was covering the detection range of one of the five primer/probe combinations. In addition one of the provided negative control strains (N1) and one NTC was used. On both cyclers the runs were performed in six technical replicates and C_t_ values were measured (Table 3).

Table 3 indicates that each of the five primer/probe combinations showed an unambiguous detection of its reference gene. All of the fluorescence curves crossed the threshold line almost in the middle of the PCR run (between cycle 13 and 16) and no amplification was detected among the negative sample (N1) and the NTC. Furthermore, no false positive fluorescence was detected in any of the four channels. Positive C_t_-signals were almost measured equally on both cyclers. In case of the Lightcycler 480II the standard deviations settled down equally around a value of 2, the CFX96 showed slightly more variation. Here, the highest standard deviation value was 4.6 (*bla*_TEM_) and the lowest was 1.3 (*bla*_CTX-M-15_). Never the less, in the next step a major sample size was analyzed in duplicates using the same settings.

### Determination of the sensitivity and specificity of the here described real-time PCR assay

Based on the investigation of the 114 previously characterized Enterobacteriaceae the obtained results possessed a sensitivity and specificity of 100%. All of the genes, preliminary identified in conventional PCR formats, were detected on the Roche Lightcycler 480II as well as on the BioRad CFX96 and none of the negative isolates turned out to be positive. The detected C_t_ values showed a slight variability but all in all they are located within a similar range. On average, all of the four fluorescence curves exceeded the cycle threshold after completion of 50 to 66% of the real-time run (between cycle 15–20; Table 4).

**Table 4:** Average over all C_t_ values and standard deviation within the analyzed gene subtypes of each beta-lactamase family detected within the 114 control strains.

		Roche Lightcycler 480II				BioRad CFX96			
		Average				Average			
		Standard deviation				Standard deviation			
*bla*- gene	n =	465–510nm	618–660nm	533–610nm	533–580nm	Channel1: FAM	Channel4: Cy5	Channel3: ROX	Channel2: VIC
negative controls	23	none				none			
AmpC_CIT_	13			15.3				16.8	
				2.1				2.3	
CTX-M groups –M1 & -M-9	29				17.7				19.6
					2.2				2.5
CTX-M groups –M-2, -M-8 & -M-25	10				19.2				20.1
					2.4				2.0
SHV	12		16.5				17.2		
			1.2				1.4		
TEM	64	16.6				17.8			
		2.2				2.3			

The runs were performed in two technical replicates and the mean C_t_ values are indicated.

n = number of tested genes.

Comparison of the C_t_ values within the analyzed gene subtypes of each beta-lactamase family showed just minor differences. The standard deviation between the measured C_t_ values are located between 1.2 (*bla*_SHV_) and 2.4 (*bla*_CTX-M_, groups -M-2/-M-8/-M-25) for the Roche Lightcycler 480II and between 1.4 (*bla*_SHV_) and 2.5 (*bla*_CTX-M_, groups -M-1 & -M-9) for the BioRad CFX96. Compared to the results shown previously for the measurements of the six technical replicates (Table 3), greater sample numbers of different subtypes of the same beta-lactamase gene family came to similar results. For both real-time cyclers and for each of the five tested primer/probe combinations almost similar standard deviations were calculated. While the *bla*_SHV_ measurements showed the best results with values of 1.2 (Roche) and 1.4 (BioRad), the standard deviations of the remaining three primer/probe combinations settled down equally around a value of 2. Moreover, no high detection-differences could be observed between strains containing just one resistance gene or the ones coding two or even three different genes within the same isolate (data not shown). Furthermore, in none of the 23 negative samples false positive fluorescence signals were detected.

However, mentionable is the fact that for seven out of 114 control strains differences between the previously provided gene subtype information and the real-time results could be traced (Table 5). During real-time assay validation one additional *bla*_SHV-11_ gene (isolate F.), two additional *bla*_TEM-52_ genes (isolates A. and B.) and three further *bla*_TEM-1_ genes (isolates C., D. and F.) were detected. In case of one isolate (E.) instead of the expected *bla*_CTX-M-9_ a *bla*_TEM-116_ appeared. All of the observed real-time results were proofed by conventional PCR and subsequent sequencing of the PCR products.

**Table 5:** Detected differences between the previously notified beta-lactamase gene subtype and the real-time results among the 114 used control strains.

Isolate	Notified beta-lactamase gene subtype	Detected beta-lactamase genes using real-time assay
A.	CMY-2	CMY-2+**TEM-52**
B.	CMY-2	CMY-2+**TEM-52**
C.	CTX-M-15	CTX-M-15+**TEM-1**
D.	CTX-M-15	CTX-M-15+**TEM-1**
E.	CTX-M-9	**TEM-116**
F.	CTX-M-26	CTX-M-26+**SHV-11**+**TEM-1**

Obtained real-time results were confirmed by conventional PCRs and sequencing of the obtained PCR products.

Additionally detected genes are marked in bolt letters.

### Assay verification using uncharacterized field strains

For the final examination of the newly described assay twenty uncharacterized field strains from different animal farms (pig, poultry) were analyzed in two technical replicates. Out of 20 investigated strains nine positive and eleven negative strains were observed. All kinds of investigated beta-lactamase genes were detected either separately or in different combinations up to four genes (Table 6). Same results were received independently in both laboratories. Like described above, the C_t_ values were located within a similar range. Finally, all of the detected beta-lactamase gene subtypes were determined via DNA-sequencing (data not shown).

**Table 6:** Summary of the different beta-lactamase gene combinations occurring among the 20 field isolates.

Detected beta-lactamase genes	Number of strains
*bla*_SHV_	2
*bla*_AmpC-CIT_	2
*bla*_SHV_ + *bla*_AmpC-CIT_	3
*bla*_AmpC-CIT_ *+ bla*_SHV_ + *bla*_TEM_	1
*bla*_CTX-M_ + *bla*_TEM_ + *bla*_SHV_ + *bla*_AmpC-CIT_	1
none of the four genes	11

## Discussion

Due to the fact that the expression of ESBL genes leads to failure of treatment with extended-spectrum cephalosporins, beta-lactamase resistant bacteria and especially ESBL producing Enterobacteriaceae are an increasing problem worldwide [1], [3]. In Europe, as well as in other non-European countries, the performance of active and passive monitoring programs on the occurrence and/or prevalence of ESBL/AmpC producing Enterobacteriaceae has been recommended [1], [13], [17] and in some countries national programs are running. For this reason efficient and reliable methods for rapid screening of high sample numbers are absolutely necessary [18].

Although there have been described other real-time PCR methods for the detection of ESBL-type beta-lactamases [19]–[22] to our knowledge, this is the first report of a multiplex real-time PCR assay for the identification of the three most common ESBL gene families plus the CIT-type AmpCs within a single run. Worth mentioning is the possibility to cope all five CTX-M subtypes within one channel of the real-time cycler. However, if a more precise differentiation is needed for a scientific question, it is still possible to label the two CTX-M probes with different fluorophores and in this way distinguish between the CTX-M types of the groups -M-1 and -M-9 on the one hand and the groups -M-2, -M-8 and-M-25 on the other hand. This is a very important fact, as during the last couple of years the CTX-M type ESBLs became the most common ESBL type worldwide [23]. As our results have shown the here described method was able to provide reliable results for all of the tested isolates. For all five primer/probe combinations and for a major set of tested isolates (min. 10 isolates for *bla*_CTX-M_, groups -M-2, -M-8 & -M-25; max. 64 isolates for *bla*_TEM_; see Table 4). The standard deviation settled around a value of 2. This observation demonstrates that the here described assay works quite stable and provides comparable results. The “proof-of-principle” by analyzing 20 previously un-typed bacterial isolates, confirmed this proposition. Beyond this, seven additional genes (6 *bla*_TEM_, 1 *bla*_SHV_) which have previously not been notified, were identified correctly (Table 5). In two cases (isolates A. and B.) additional *bla*_TEM-52_ genes have been detected. As *bla*_TEM-52_ belongs to the 2be phenotype of ESBL genes (http://www.lahey.org/Studies/temtable.asp), this information could have been crucial. In case of three strains the *bla*_CTX-M_ subtypes were identified within the preliminary analyses; never the less additional *bla*_TEM-1_ or *bla*_SHV-11_ genes were detected. However, even if this lack of identification would not cause serious problems in case of antimicrobial therapy, regarding epidemiological purposes genes leading to the 2b phenotype are noteworthy as well. Last but not least for the fifth strain (E.) the real-time PCR gave the hint that two strains might have been mixed up here. Instead of the expected *bla*_CTX-M-9_ the analyzed strain turned out to carry a *bla*_TEM-116_. However, of same importance as speed and precision is the reproducibility in different laboratories. Only if a method is working on different machines and in different surroundings with the same accuracy it will be a good tool for the use in laboratory practice. Like to be shown, the multiplex real-time method fulfills these requirements without any difficulties. Comparing the here described method - containing a running time of just one hour - with conventional PCR techniques and subsequent gel electrophoresis, the real-time assay provides a cheaper and much faster service. Even if sequence analyses are further necessary for detailed characterization of the gene subtypes, the accurate and prompt identification of the resistance genes will be an important tool for the use in various scientific fields (e.g. epidemiological purposes, medical issues, etc.). Particularly with regard to screening analyses, describing the *status-quo* of resistance gene distribution within or between different environments, the here described real-time assay could serve as a highly effective method for the detection/confirmation of ESBL/CIT-type AmpC genes.
